# Green Tea Extract Containing a Highly Absorbent Catechin Prevents Diet-Induced Lipid Metabolism Disorder

**DOI:** 10.1038/srep02749

**Published:** 2013-09-25

**Authors:** Takashi Suzuki, Motofumi Kumazoe, Yoonhee Kim, Shuya Yamashita, Kanami Nakahara, Shuntaro Tsukamoto, Masako Sasaki, Takatoki Hagihara, Yukari Tsurudome, Yuhui Huang, Mari Maeda-Yamamoto, Yuki Shinoda, Wataru Yamaguchi, Koji Yamada, Hirofumi Tachibana

**Affiliations:** 1 Division of Applied Biological Chemistry, Department of Bioscience and Biotechnology, Faculty of Agriculture, Kyushu University, Fukuoka 812-8581, Japan; 2 National Food Research Institute, National Agriculture and Food Research Organization, Ibaraki 306-8642, Japan; 3 Products Research & Development Laboratory, Asahi Soft Drinks Co, Ltd., Ibaraki, 302-0106, Japan; 4 Food Functional Design Research Center, Kyushu University, Fukuoka 812-8581, Japan

## Abstract

We investigated the effects of extracts of Benifuuki (a tea cultivar that contains methylated catechins such as epigallocatechin-3-*O*-(3-*O*-methyl) gallate (EGCG3”Me)) in mice fed a high-fat/high-sucrose (HF/HS) diet. This tea cultivar was then compared with an extract of Yabukita (a popular tea cultivar that lacks methylated catechins). For 6 weeks, C57BL/6J mice were fed either HF/HS diet with or without tea extracts from tea cultivars, which contained almost identical ingredients except for methylated catechins (i.e., Yabukita (0.2% and 1%) or Benifuuki (0.2% and 1%) extract powders). Supplementation with Benifuuki 0.2% markedly lowered plasma levels of TG and NEFAs compared with mice supplemented with Yabukita 0.2%. The diet containing Benifuuki 1% decreased adipose tissue weights, liver TG, and expression of lipogenic genes in the liver. These results suggested that Benifuuki had much greater lipid-lowering effects than Yabukita. Taken together, these data suggest that methylated catechins direct the strong lipid-lowering activity of Benifuuki.

Obesity is a metabolic disturbance resulting from an imbalance between fat synthesis (lipogenesis) and fat breakdown (oxidation). Obesity is prevailing at an alarming rate worldwide. Obesity is associated with an increased risk of cardiovascular disease^1^. Indeed, obesity-induced hypercholesterolaemia is correlated with coronary heart disease risk^2^.

Green tea (*Camellia sinensis* L.) is one of the most popular beverages in the world. It has been reported that daily consumption of green tea is associated with several important health benefits, including antioxidant, anti-carcinogenic, hypocholesterolemic, and cardioprotective activities^3^,^4^,^5^,^6^. The beneficial properties of green tea are attributable to the abundant polyphenolic compounds (“catechins”) that it contains. The green-tea catechins include catechin (C), (−)-epicatechin (EC), (−)-epigallocatechin (EGC), (−)-epicatechin-3-gallate (ECG), and (−)-epigallocatechin-3-gallate (EGCG). Of these, EGCG is a predominant catechin, and has several biological and pharmacological properties^7^,^8^,^9^,^10^,^11^,^12^,^13^,^14^,^15^,^16^. Several studies have reported the anti-obesity effects of green tea and EGCG^17^,^18^,^19^,^20^.

Benifuuki (*Camellia sinensis* L.) is a tea cultivar that was originally established by the National Institute of Vegetable and Tea Science (National Agriculture and Food Research Organization, Tokyo, Japan). Benifuuki is rich in methylated catechins such as epigallocatechin-3-*O*-(3-*O*-methyl) gallate (EGCG3”Me; ≈ 0.8–2.5% (*w/w*)), which is not present in Yabukita cultivar, the most popular green tea, which accounts for about 75% of green-tea products consumed in Japan. *In vivo* and clinical studies have shown that Benifuuki has strong anti-allergic effects^21^,^22^. Pharmacokinetic analyses confirmed that EGCG3”Me has a significantly higher absorption rate from the intestine and a lower rate of disappearance from the blood than EGCG^23^. Therefore, although the amount of EGCG3”Me in Benifuuki tea is about one-quarter that of EGCG, the blood concentrations of these two catechins after ingestion of Benifuuki tea are almost identical^23^. This is considered to be the main reason why Benifuuki has greater anti-allergic activity than Yabukita. The anti-allergic effects of Benifuuki have been reported, but studies describing the beneficial anti-obesity effects of Benifuuki have not.

The goals of the present study were to determine the effect of dietary Benifuuki on fat accumulation, lipid levels in the plasma and liver, and hepatic gene expression in a HF/HS-induced model of obesity in mice.

## Results

### General observations

Benifuuki is rich in methylated catechins such as EGCG3”Me, which is not present in Yabukita, the common cultivar. To determine the effect of dietary Benifuuki on fat accumulation, lipid levels in the plasma and liver, and hepatic gene expression in a HF/HS-induced model of obesity and metabolic syndrome in mice, mice were given Yabukita or Benifuuki extract (Table 1) as described.

**Figure 1:**
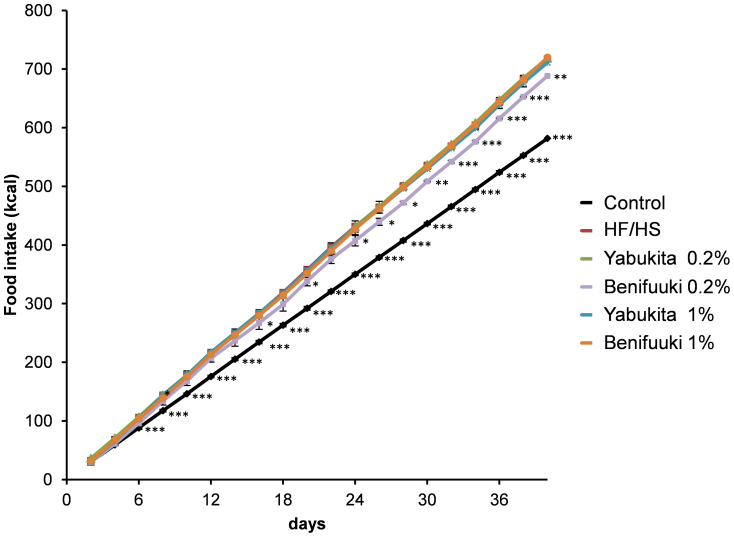
Food intake. Food intake was measured on a per-cage basis throughout the study and represents cumulative energy intake. Values are means ± SEM. (vs HF/HS-fed group) *p < 0.05. **p < 0.01. ***p < 0.001. *n* = 6.

HF/HS feeding for 6 weeks significantly increased body weight and the weight of retroperitoneal adipose tissue compared with a control diet (Table 2). The groups fed Yabukita 1% and Benifuuki 1% had moderate decreased in body weight and weight of retroperitoneal adipose tissue compared with the HF/HS-fed group. The groups fed Yabukita 1% and Benifuuki 1% had a moderate decrease in body weight and weight of retroperitoneal adipose tissue compared with the HF/HS-fed group. The groups fed Benifuuki 1% had a moderate decrease in both rWAT and eWAT. However, the group fed Yabukita 1% had a moderate decrease in only eWAT. The cumulative energy intake did not significantly differ among the HF/HS group and the tea-extract groups except Benifuuki 0.2% group (Fig. 1).

### Biochemical analyses of plasma

**Figure 2:**
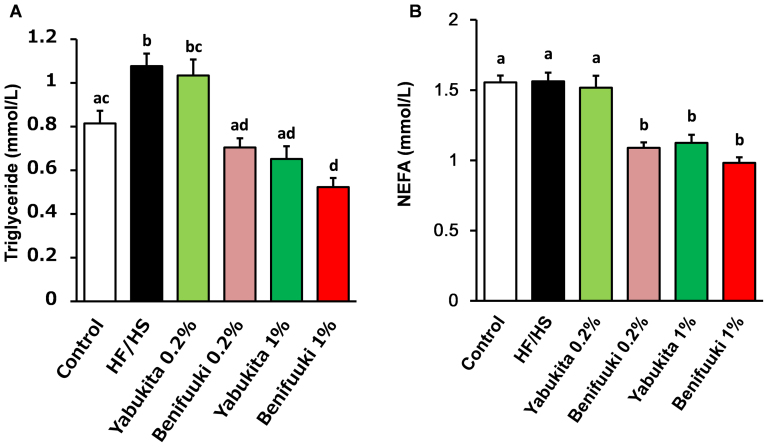
Effects of Yabukita and Benifuuki on serum levels of TG and NEFA in HF/HS-fed mice. Plasmsa TG (A) and NEFA (B) at the end of treatment period. Values are means ± SEM, *n* = 6. Different letters are statistically different, *p* < 0.05.

HF/HS feeding significantly elevated levels of total cholesterol (TC), high-density lipoprotein cholesterol (HDL-C), low-density lipoprotein cholesterol (LDL-C) and very-high density lipoprotein cholesterol (VLDL-C) (Table 2). A Benifuuki 1% diet significantly reduced levels of LDL-C and VLDL-C. Yabukita 1% as well as Benifuuki 0.2% and 1% diets significantly decreased plasma levels of aspartate aminotransferase (AST) and alanine transaminase (ALT). HF/HS feeding also elevated plasma levels of TG (Fig. 2A). A Yabukita 0.2% diet did not affect plasma levels of TG, whereas Yabukita 1% as well as Benifuuki 0.2% and 1% diets lowered plasma levels of TG to those seen in controls. Yabukita 1% as well as Benifuuki 0.2% and 1% diets lowered plasma levels of non-esterified fatty acid (NEFA) (Fig. 2B).

### Liver lipids

**Figure 3:**
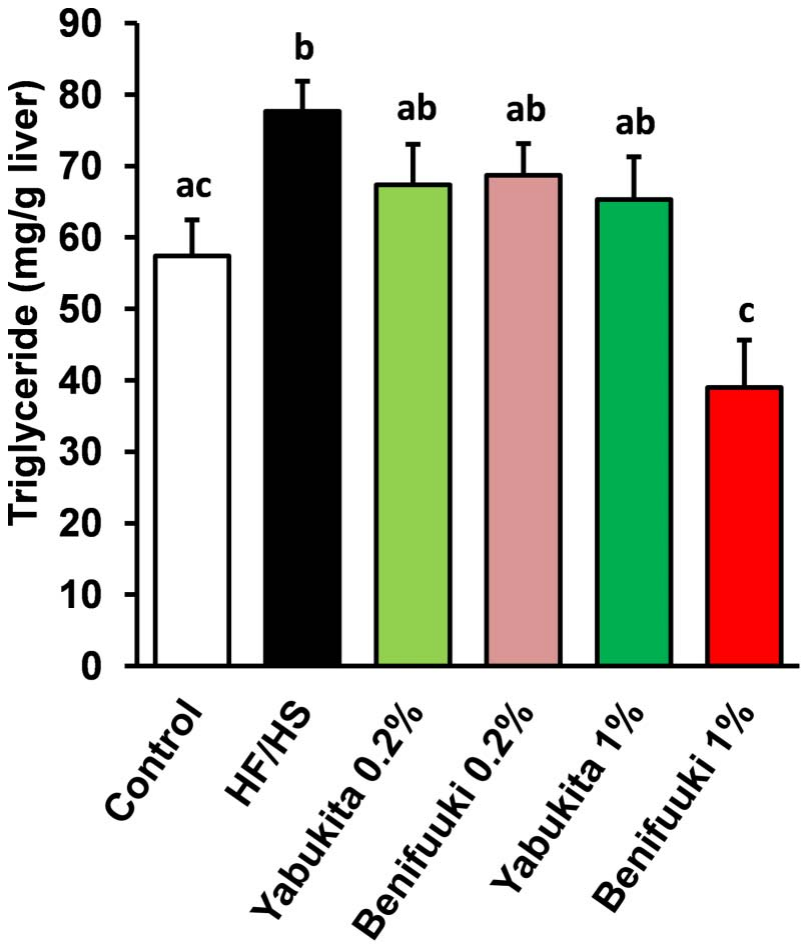
Effects of Yabukita and Benifuuki on liver levels of TG in HF/HS-fed mice. Liver TG at the end of treatment period. Values are means ± SEM, *n* = 6. Different letters are statistically different, *p* < 0.05.

HF/HS feeding for 6 weeks increased the content of liver TG. A Benifuuki 1% diet had a profound lowering effect upon liver levels of TG (Fig. 3).

### DNA chip analyses

Among 194 genes associated with the metabolism of lipids and carbohydrates, we used data for the other 121 genes for analyses in the present study because the expressions of 73 of these genes were barely detected in liver samples (Supplementary Table S1 and S2). DNA chip analyses revealed that HF/HS, Yabukita, and Benifuuki diets had a wide influence on mRNA expression. The diets affected the genes linked to energy production, redox regulation, defense against reactive oxygen species (ROS), the mitogen-activated protein kinase (MAPK) cascade, nuclear receptors, metabolism of energy and cholesterol, and protein degradation. In particular, the expression of genes related to the metabolism of energy and cholesterol was influenced by the Benifuuki diet (Supplementary Table S1 and S2).

### Expression of genes involved in the metabolism of lipids and cholesterol

**Figure 4:**
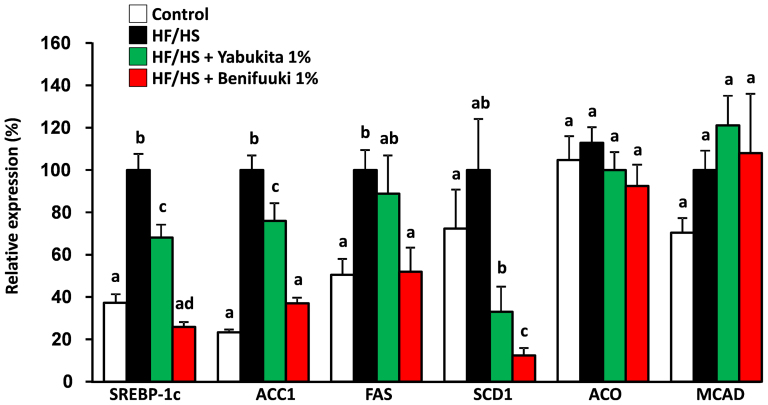
Effects of Yabukita and Benifuuki on mRNA levels of genes involved in lipid metabolism in liver. The mRNA expression levels of genes involved in TG synthesis (SREBP-1c, ACC1, FAS and SCD1) and fatty acid metabolism (ACO and MCAD) in the liver of C57/BL6J mice fed diets containing HF/HS and Yabukita 1% or Benifuuki 1% extract powders. Values are means ± SEM, *n* = 6. Different letters are statistically different, *p* < 0.05.

**Figure 5:**
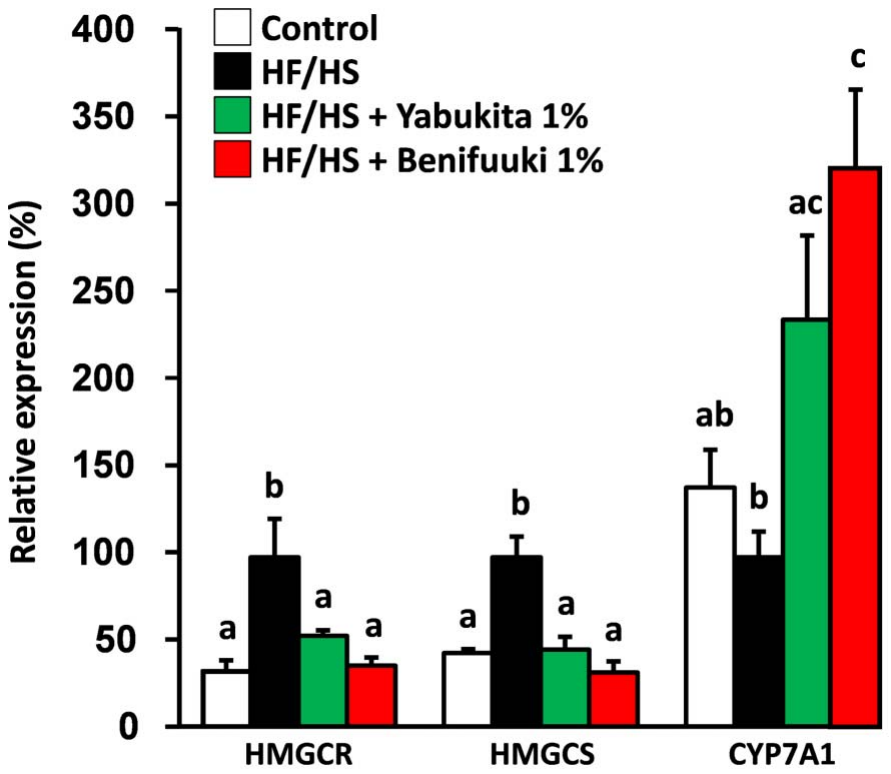
Effects of Yabukita and Benifuuki on mRNA levels of genes involved in cholesterol metabolism in liver. The mRNA expression levels of genes involved in cholesterol metabolism (HMGCR, HMGCS and CYP7A1) in C57/BL6J mice fed diets containing HF/HS and Yabukita 1% or Benifuuki 1% extract powders. Values are means ± SEM, *n* = 6. Different letters are statistically different, *p* < 0.05.

We examined the expression of hepatic genes that regulate lipogenesis, beta-oxidation and cholesterol metabolism to explore the molecular mechanism underlying the hypolipidemic effect of a Benifuuki diet by real-time PCR. A Benifuuki diet decreased the expression of genes associated with lipogenesis such as sterol regulatory element binding protein-1c (SREBP-1c), acetyl-CoA carboxylase1 (ACC1), fatty acid synthase (FAS) and stearoyl-CoA desaturase1 (SCD1), compared with the HF/HS-fed group (Fig. 4). HF/HS feeding increased the expression of hepatic 3-hydroxy-3methyl-glutaryl-CoA reductase (HMGCR) and 3-hydroxy-3-methylglutaryl-CoA synthase (HMGCS), whereas Yabukita 1% and Benifuuki 1% diets lowered their expressions to those seen in controls. HF/HS did not affect the expression of cholesterol 7-alpha-monooxygenase (CYP7A1), whereas Yabukita 1% and Benifuuki 1% diets increased the expression of CYP7A1 mRNA (Fig. 5).

## Discussion

In the present study, we showed that Benifuuki (a tea cultivar that contains methylated catechins) had a stronger lowering effect on plasma and hepatic TGs in HF/HS diet-fed mice than Yabukita (which lacks methylated catechins).

Mice fed Benifuuki 1% had reduced adipose tissue weights and liver TG content. Yabukita showed a less potent effect on TG content. These results suggested that Benifuuki had much greater hypolipidemic activity and anti-obesity effects than Yabukita. A marked difference in the total energy intake between HF/HS, Yabukita 1% and Benifuuki 1% groups was not observed. Therefore, various mechanisms leading to increased energy expenditure or lower fat deposition might be involved in the effects elicited by Benifuuki. Dietary HF/HS is a strong inducer of SREBP1-c, FAS, SCD1, and other enzymes regulating hepatic lipogenesis, and produces a pronounced elevation of hepatic TG concentrations with an increase in plasma TG^24^,^25^,^26^,^27^,^28^,^29^. Benifuuki downregulated the expression of lipogenic enzyme genes such as SREBP-1c, ACC1, FAS and SCD1 in the liver. Several lines of evidence suggest that the suppression or disruption of ACC1 (a cytosolic enzyme that catalyzes the carboxylation of acetyl-CoA to form malonyl-CoA) leads to the reduction of the synthesis and accumulation of hepatic TGs^30^, suggesting that suppression of ACC1 expression may be due to the reduction of hepatic fat accumulation in Benifuuki-fed mice.

FAS catalyzes the last step in the biosynthetic pathway of fatty acids. Hence, it is believed to be a determinant of the maximal capacity of a tissue (the liver in particular) to synthesize fatty acids by *de novo* lipogenesis. Increases in FAS levels are attributed to elevation of serum levels of TG and NFEAs as well as liver TG^31^. Hence, downregulation of FAS expression might lead to a reduction in lipid levels in the liver and serum in Benifuuki-fed mice. SCD1 (which catalyzes the biosynthesis of monounsaturated fatty acids from saturated fatty acids) also has an important role in energy metabolism and regulation of body weight^32^. Importantly, Benifuuki intake significantly downregulated SCD1 expression. SCD1 plays an essential role in monounsaturated fatty acids for hepatic lipogenesis/TAG synthesis^33^. Treatment of mice with SCD1 antisense oligonucleotides has been shown to result in a higher metabolic rate, prevention of diet-induced obesity, and steatosis^34^. Therefore, decreased SCD1 expression could explain the reduction in body fat observed in Benifuuki-fed mice. The expression of lipogenic genes such as ACC1, FAS and SCD1 is regulated by SREBP-1c at the transcriptional level^35^,^36^. Our data suggested that Benifuuki suppresses the expression of SREBP-1c and results in the reduction of hepatic lipogenic genes (ACC1, FAS and SCD1). Yabukita and Benifuuki were not influenced on genes related to the oxidation of fatty acids (e.g., ACO and MCAD). Taken together, a plausible mechanism for the hypolipidemic activity of Benifuuki may be its downregulation of gene expression associated with lipid synthesis and not with fatty-acid oxidation.

The upregulation of hepatic HMGCS and HMGCR mRNA by HF/HS is consistent with the increased concentration of cholesterol in plasma. Our data showed that green tea suppresses genes associated with cholesterol synthesis (HMGCS, HMGCR) and upregulates CYP7A1 expression in the liver. Yabukita and Benifuuki, however, were ineffective in preventing the HF/HS-induced increase in concentrations of TC in plasma. The effects of green tea on suppression of the genes that regulate cholesterol metabolism were unrelated to TC accumulation in plasma. The reduction of plasma levels of LDL-C/VLDL-C in Benifuuki-fed mice might have been due to suppression of the expression of lipogenesis genes.

Many of the beneficial effects of green tea are considered to be mediated by tea catechin, especially EGCG (the most abundant catechin found in green tea). Several studies have shown that EGCG has anti-obesity effects^37^,^38^. There are no significant differences in catechin composition between Benifuuki and Yabukita extracts except for methylated catechins. EGCG3”Me constitutes 6.8% of the total catechins in Benifuuki. It has been reported that if Benifuuki extracts are administered orally to humans, the area under the drug concentration–time curve (AUC) of EGCG3”Me is higher than that of EGCG even though the dose of EGCG is 5.1-times the dose of EGCG3”Me^21^. Clinical trials have confirmed that EGCG3”Me is absorbed six-times more quickly than EGCG in humans and that it disappears more slowly from the blood than EGCG^21^. The level of EGCG plus EGCG3”Me in plasma from Benifuuki-administered mice was 5.2 times higher than levels in Yabukita-administered mice. However, the content of EGCG3”Me in Benifuuki was about one-quarter of EGCG's content (Supplementary Fig. 1). It is unlikely that a single substance is responsible for its many beneficial effects because green tea contains many substances. However, with regard to the greater hypolipidemic effect of Benifuuki than Yabukita, EGCG3”Me is probably play a essential role in its bioactivity because large amounts are present in Benifuuki but not in Yabukita. The safety of Benifuuki has been confirmed the result of serum ALT and AST. Furthermore, there are no differences in the parameters for the analyses of blood and urine, pathological examination, or subjective symptoms before and after clinical trials involving long-term treatment with Benifuuki^21^.

In summary, the present study provides new evidence that tea containing unique methylated catechins such as EGCG3”Me significantly downregulates the hepatic expression of lipid-synthesis genes such SREBP1-c, ACC1, FAS and SCD1. Based on the data and information available, the TG-lowering effect of Benifuuki in plasma and the liver may be mediated (at least in part) *via* the suppression of lipogenesis, probably because of the high absorption rate and stability of EGCG3”Me in plasma and its effect on lipogenesis genes. However, studies focusing on whether EGCG3”Me has lipid-lowering effects *in vivo* and *in vitro* are lacking. Therefore, further studies are needed to better understand the mechanism of Benifuuki and EGCG3”Me.

## Methods

### Mice and diet

Male C57/BL6 mice (12 weeks) were purchased from Charles River (Kanagawa, Japan). They were maintained in a temperature- and humidity-controlled room with a 12-h-light–dark cycle (light from 8 am to 8 pm). All mice were acclimated for 1 week while being fed an AIN-93G diet. AIN-93G and HF/HS diets were obtained from KBT Oriental (Tokyo, Japan) (Table 3).

Mice were assigned to six groups: (1) a negative control group (control) fed a AIN-93G diet; (2) a positive control group (HF/HS) fed a high-fat/high-sucrose diet; (3) a group (Yabukita 0.2%) fed the HF/HS diet but containing 0.2% Yabukita; (4) a group (Yabukita 1.0%) fed a HF/HS diet containing 1.0% Yabukita; (5) a group (Benifuuki 0.2%) fed a HF/HS containing 0.2% Benifuuki; and (6) a group (Benifuuki 1.0%) fed the HF/HS diet containing 1.0% Benifuuki.

Extracts of Yabukita and Benifuuki were prepared from the leaves of the plants using boiling water followed by freeze-drying. The composition of each extract powder was determined by high-performance liquid chromatography (HPLC) (Table 1). The dietary levels of green tea at 0.2% and 1.0% were equivalent to human intakes of 1.6 cups and 8 cups (2.0 g of green tea/cup) per day, respectively, as estimated based on an energy intake of 8,360 kJ/day. Mice were fed the diets three times a week and were weighed every week. At the end of 6 weeks of feeding, mice were anesthetized under isoflurane vapor after overnight food deprivation. Blood samples were collected into tubes *via* the retro-orbital sinus. Plasma was collected after centrifugation (2000 × *g* for 20 min at 4°C) and stored at −80°C. After blood collection, mice were killed by isoflurane overdose. Visceral adipose tissues (peritoneal and epididymal depots) and livers were harvested, rinsed, and weighed. From the central lobe of the liver, ~ 0.5 g tissue was excised from each mouse and placed in a plastic tube containing RNALater™ solution (Ambion, Austin, TX, USA). Liver tissue in RNALater was maintained at 4°C for 24 h and then stored at −80°C. This experiment was carried out according to the guidelines for animal experiments at the Faculty of Agriculture, Kyushu University. The study protocol was approved by the Animal Care and Use Committee of Kyushu University, Fukuoka, Japan. The approval number for the animal experiment is A22-146.

### Measurement of the amount of catechins and caffeine in tea extracts

Tea samples were prepared for HPLC analyses. Briefly, samples were immersed in 50% ethanol containing 1% H_3_PO_4_, disrupted using ultrasonic apparatus for 30 min at 20°C, and then filtered through a membrane filter (pore size, 0.45 μm). HPLC was undertaken using a Shimadzu LC-10A pump coupled to an ultraviolet detector (SPD-M10Avp; Shimadzu, Kyoto, Japan) and a reverse-phase Wakopak Navi C18-5 column (4.6 mm i.d. × 150 mm; granule diameter, 5 μm; Wako Pure Chemical Industries, Kyoto, Japan). Elution consisted of a 2−45-min linear gradient from 0 to 80% of solvent B and solvent A. Solvent A consisted of distilled water/phosphoric acid/acetonitrile (400:10:1 *v/v*), and solvent B consisted of methanol/solvent A (1:2 *v/v*). Samples were eluted at 1 mL/min at 40°C. The detection wavelength was 272 nm. The amounts of catechins and caffeine in tea extracts were measured by comparing the peak area of each catechin in the tea extract with that of a standard preparation that contained a fixed quantity of caffeine, EC, C, EGCG, gallocatechin gallate (GCG), ECG, catechin gallate (CG), EGCG3”Me, and gallocatechin-3-*O*-(3-*O*-methyl) gallate (GCG3″Me).

### Biochemical analyses of plasma and liver samples

Plasma levels of triglyceride (TG), non-esterified fatty acids (NEFAs), aspartate aminotransferase (AST), alanine transaminase (ALT), total cholesterol (TC), high-density lipoprotein cholesterol (HDL-C), low-density lipoprotein-cholesterol (LDL-C) and very low-density lipoprotein-cholesterol (VLDL-C) were measured using the TG E-test, the non-esterified fatty acid (NEFA) C-test and the transaminase CII-test (each from Wako), HDL-C and LDL-C/VLDL-C Quantification Kit (BioVision, Milpitas, CA, USA), respectively. The liver TG content were measured using the TG E-test (from Wako), respectively, after the extraction of hepatic lipids with chloroform-methanol (2:1). All kits were used in accordance with manufacturer instructions.

### RNA isolation and DNA chip analyses

Total RNA was extracted from liver samples using the RNeasy™ Mini Kit (Qiagen, Valencia, CA, USA). All total RNA samples were analyzed using an Agilent 2100 Bioanalyzer (Agilent Technologies, Palo Alto, CA, USA). Biotinylated antisense RNA was synthesized and amplified from total RNA (1 μg) using the MessageAmpII Biotin Enhanced Amplification kit (Applied Biosystems, Foster City, CA, USA) according to manufacturer protocols. After aRNA purification, biotinylated aRNA (5 μg) was fragmented using 10 × fragmentation reagents (Applied Biosystems) by heating at 70°C for 7.5 min. Hybridization was carried out with DNA chips in 150 μL hybridization buffer (0.12 M Tris HCl/0.12 M NaCl/0.05% Tween-20 and 5 μg fragmented biotinylated aRNA) at 65°C overnight. After hybridization, DNA chips were washed twice in 0.12 M Tris HCl/0.12 M NaCl/0.05% Tween-20 at 65°C for 20 min, followed by washing in 0.12 M Tris HCl/0.12 M NaCl for 10 min. Then hybridized aRNA was labeled with 2 μg/mL streptavidin-Cy5 (GE Healthcare, Little Chalfont, UK) in 0.12 M Tris HCl/0.12 M NaCl for 30 min at room temperature. After fluorescent labeling, DNA chips were washed four times in 0.12 M Tris HCl/0.12 M NaCl/0.05% Tween-20 at room temperature for 5-min each. DNA chips were scanned at multiple exposure times (0.1–40 s) using a DNA Chip Reader (Yokogawa Electric, Tokyo, Japan) with multi-beam excitation technology. The intensity values with the best exposure condition for each spot were selected. The median value of background spots was subtracted from the intensity value in each gene, and thereafter the value was normalized to the expression of an endogenous control (acidic ribosomal phosphoprotein, beta-actin and glyceraldehyde-3-phosphate dehydrogenase.

### Real-time quantitative polymerase chain reaction (PCR)

cDNA was synthesized from total RNA (1 μg) using the PrimeScript RT reagent Kit (Takara Bio, Tokyo, Japan). Gene expression was analyzed by real-time quantitative PCR using the Sybr green procedure and a Thermal Cycler Dice Real Time System (Takara Bio). Primer sequences are given in Supplementary Table S2. Expression of mRNA values was normalized relative to glyceraldehyde-3-phosphate dehydrogenase as an internal control.

### Statistical analyses

All statistical analyses were performed using one-way ANOVA with Tukey's *post hoc* test using Kyplot software. Data are the means ± SEM. P < 0.05 was considered significan.

## Author Contributions

T.S. M.K. T.H. K.Y. and H.T. designed the research. T.S. M.K. Y.K. S.Y. S.T. M.S. K.N. T.H. Y.T. S.Y. Y.H. K.N. M.M. Y.S. W.Y. K.J. and H.T. conducted the research. S.T. T.H. Y.T. and H.T. analyzed data and undertook statistical analyses. T.S. and H.T. wrote the manuscript. H.T. had primary responsibility for the final content. All authors approved the final manuscript for submission.

## Supplementary Material

Supplementary Information Supplementary Figure 1, Supplemental table 1,Supplemental table 2

## Figures and Tables

**Table 1:** Composition of tea extract powders (mg/g)

	Yabukita extract powder (mg/g)	Benifuuki extract powder (mg/g)
galocatechin (GC)	24.4	24
(−)-epigallocatechin (EGC)	81.8	76.3
catechin (C)	14.7	6.6
(−)-epicatechin (EC)	22.3	27.8
(−)-epigallocatechin-3-gallate (EGCG)	134.2	108.5
gallocatechin gallate (GCG)	21.4	19.1
(−)-epicatechin-3-gallate (ECG)	24.8	28.5
catechin gallate (CG)	2.5	2.9
(−)-epigallocatechin-3-*O*-(3-*O*-methyl) gallate (EGCG”3Me)	0	21.6
gallocatechin-3-*O*-(3-*O*-methyl) gallate (GCG”3Me)	0	4.7
Methylated catechins (EGCG”3Me + GCG”3Me)	0	26.3
Total catechins	326.2	320.1

**Table 2:** Effects of Yabukita and Benifuuki on accumulation of body fat and serum components in HF/HS-fed C57BL/6J mice

			HF/HS + Yabukita		HF/HS + Benifuuki	
	Control	HF/HS	0.2%	1%	0.2%	1%
Final body weight, g	30.70 ± 0.68^ac^	35.75 ± 0.95^b^	35.22 ± 0.58^b^	31.12 ± 1.24^ac^	34.15 ± 1.01^abc^	29.00 ± 0.78^c^
Perirenal fat, g	0.39 ± 0.03^ac^	0.67 ± 0.05^b^	0.67 ± 0.04^b^	0.49 ± 0.09^ab^	0.62 ± 0.04^b^	0.21 ± 0.07^c^
Epididymal fat, g	0.99 ± 0.09^ac^	1.91 ± 0.16^b^	1.73 ± 0.11^b^	1.08 ± 0.16^ac^	1.45 ± 0.10^ab^	0.64 ± 0.17^c^
Liver, g	1.01 ± 0.02^ab^	1.09 ± 0.09^a^	1.10 ± 0.04^a^	0.93 ± 0.06^bc^	1.03 ± 0.06^ab^	0.88 ± 0.07^c^
LDL/VLDL cholesterol, mmol/l	0.64 ± 0.15^a^	1.17 ± 0.18^bc^	1.21 ± 0.20^b^	0.85 ± 0.08^ab^	0.92 ± 0.08^ab^	0.69 ± 0.13^ac^
HDL cholesterol, mmol/l	3.08 ± 0.21	4.03 ± 0.17	4.02 ± 0.43	3.85 ± 0.20	3.70 ± 0.18	3.49 ± 0.35
Total cholesterol, mmol/l	3.55 ± 0.22	4.62 ± 0.22	4.50 ± 0.26	4.31 ± 0.19	4.12 ± 0.20	3.60 ± 0.50
AST, U/l	30.9 ± 2.1^a^	30.8 ± 0.7^a^	26.8 ± 1.3^ab^	19.5 ± 1.1^b^	21.1 ± 0.8^b^	19.0 ± 4.5^b^
ALT, U/l	13.2 ± 0.8^a^	12.2 ± 0.9^a^	10.0 ± 1.2^ab^	6.7 ± 1.3^b^	7.1 ± 1.4^b^	2.8 ± 0.5^b^

Values are means ± SEM, *n* = 6. Labeled means at a time without a common letter differ, *p* < 0.05.

**Table 3:** Composition of experimental diets

			HF/HS + Yabukita		HF/HS + Benifuuki	
	Control	HF/HS	0.2%	1%	0.2%	1%
Macronutrient composition						
Protein,% of energy	17.9	21.6	21.6	21.6	21.6	21.6
Fat,% of energy	7	30.3	30.3	30.3	30.3	30.3
Energy, MJ/kg	15.8	21.1	21.1	21.1	21.1	21.1
Ingredient (g/kg)						
Vitamin mix	10	10	10	10	10	10
Mineral mix	35	35	35	35	35	35
Choline bitartrate	2.5	2.5	2.5	2.5	2.5	2.5
L-Cystin	3	3.8	3.8	3.8	3.8	3.8
Soybean oil	70	20	20	20	20	20
Tertiary butylhydroquinone	0.01	0.06	0.06	0.06	0.06	0.06
Sucrose	100	200	200	200	200	200
Casein	200	250	250	250	250	250
Corn strach	132	148.7	148.7	148.7	148.7	148.7
Pegelatinized corn starch	397.5	0	0	0	0	0
Cellulose	50	50	48	40	48	40
Tallow	0	140	140	140	140	140
Lard	0	140	140	140	140	140
Yabukita extract powder	0	0	2	10	0	0
Benifuuki extract powder	0	0	0	0	2	10

Vitamin mix contains the following (g/kg vitamin mix): all-*trans*-retinol acetate, 0.80; cholecalciferol, 0.25; all-rac-α-tocopherol acetate, 15; d-biotin, 0.20; cyanocobalamin (0.1%), 2.5; folic acid, 0.20; Ca-panthothenate, 1.6; niacin, 3.0; pyridoxine-HCI, 0.70; thiamin-HCl, 0.60; riboflavin, 0.60; phylloquinone, 7.5 and sucrose, 974.66. Mineral mix contains the following (g/kg mineral mix): magnesium oxide, 24; calcium carbonate, 357; potassium phosphate monobasic, 250; tripotassium citrate monohydrate. 28; sodium chloride. 74; potassium sulfate. 46.6; iron citrate. 6.06; zinc carbonate. 1.65; manganese carbonate. 0.63; copper carbonate basic. 0.324; potassium Iodate, 0.01; sodium selenate. 0.01; ammonium molybdate,4H_2_O 0.008; sodium silicate.9H_2_O, 0.0145; chromium potassium sulfate.12H_2_O, 0.275; lithium chloride, 0.0174; boric acid, 0.0815; sodium fluoride, 0.0635; nickel carbonate basic 4H_2_O, 0.0306; zmmonium metavanadate, 0.0066; and sucrose, 209.78.
