# Supplementary material for: Green Tea Extract Containing a Highly Absorbent Catechin Prevents Diet-Induced Lipid Metabolism Disorder
Source: Sci Rep. 2013 Sep 25;3:2749. doi: 10.1038/srep02749 (PMC3782887; doi:10.1038/srep02749)
Supplement: Supplementary Information — Supplementary Figure 1, Supplemental table 1,Supplemental table 2 [file srep02749-s1.docx]

**Supplementary information**

**Green Tea Extract Containing a Highly Absorbent Catechin Prevents Diet-Induced Lipid Metabolism Disorder.**

Takashi Suzuki^1^, Motofumi Kumazoe^1^, Yoonhee Kim^1^, Shuya Yamashita^1^, Kanami Nakahara^1^, Shuntaro Tsukamoto^1^, Masako Sasaki^1^, Takatoki Hagihara^1^, Yukari Tsurudome^1^, Yuhui Huang^1^, Mari Maeda-Yamamoto^2^, Yuki Shinoda^3^, Wataru Yamaguchi^3^, Koji Yamada^1^ and Hirofumi Tachibana^1,4^

*^1^ Division of Applied Biological Chemistry, Department of Bioscience and Biotechnology, Faculty of Agriculture, Kyushu University, Fukuoka 812-8581, Japan*

*^2^ National Food Research Institute, National Agriculture and Food Research Organization*

*^3^ Products Research & Development Laboratory, Asahi Soft Drinks Co, Ltd., Ibaraki, Japan*

*^4^ Functional Food Design Research Center, Kyushu University, Fukuoka 812-8581, Japan*

* E-mail: tatibana@agr.kyushu-u.ac.jp

**Supplementary information**

**Supplementary Figure S1. Quantitative analysis of EGCG and EGCG3ˮMe in orally Yabukita- or Benifuuki- administrated mouse plasma.** The amount of EGCG and EGCG3ˮMe in mouse plasma was determined by LC-MS analysis at the 1h after administration. The data are expressed as the mean ± SEM and data were analyzed using the unpaired t-test. Mean values were significantly different from those of the Yabukita at *p* < 0.05. n = 5. n.s.; not significant, ***p* < 0.01

**LC-MS analysis of EGCG and EGCG3”Me in plasma samples.**

Male C57/BL6 mice (13 weeks, five per group) were given a single dose of Yabukita or Benifuuki extract (50 mg/mouse) as an aqueous solution with sterile saline and then sacrificed 1 h after administration. Mouse plasma was collected and mixed with equal volume of the ethyl acetate. The ethyl acetate fractions of plasma were evaporated to dryness in a vacuum centrifuge concentrator. Samples were reconstituted in 10 % aqueous ACN with 10 mM 4-hydroxybenzophenone as an internal standard. Plasma extracts were subjected to ESI-LC-MS analysis using a LCMS-IT-TOF instrument (Shimadzu). The instrument was fitted with a Luna 5u C18 (2) 100A column (250 × 1.0 mm, 5 µm, Phenomenex). The oven temperature was 40 °C. The mobile phase was a binary gradient of solvent A (H2O containing 0.1 % formic acid) and solvent B (MeOH). Solvent B was increased from 5 % to 60 % over 2 min, and then increased from 60 % to 100 % at 14 min. The mobile phase flow rate was 0.1 ml/min. Samples were filtered through a 0.2 µm polytetrafluoroethylene filter, and 12 μL was injected. The MS instrument was operated using an ESI source in negative ionization mode with survey scans acquired from m/z 100 to 800. Quantification was carried out using the external standard method.
